# Visualising the global structure of search landscapes: genetic improvement as a case study

**DOI:** 10.1007/s10710-018-9328-1

**Published:** 2018-08-06

**Authors:** Nadarajen Veerapen, Gabriela Ochoa

**Affiliations:** Computing Science and Mathematics, University of Stirling, Stirling, FK9 4LA UK

**Keywords:** Fitness landscape, Local optima network, Genetic improvement, Visualisation

## Abstract

The search landscape is a common metaphor to describe the structure of computational search spaces. Different landscape metrics can be computed and used to predict search difficulty. Yet, the metaphor falls short in visualisation terms because it is hard to represent complex landscapes, both in terms of size and dimensionality. This paper combines local optima networks, as a compact representation of the global structure of a search space, and dimensionality reduction, using the t-distributed stochastic neighbour embedding algorithm, in order to both bring the metaphor to life and convey new insight into the search process. As a case study, two benchmark programs, under a genetic improvement bug-fixing scenario, are analysed and visualised using the proposed method. Local optima networks for both iterated local search and a hybrid genetic algorithm, across different neighbourhoods, are compared, highlighting the differences in how the landscape is explored.

## Introduction

Fitness landscapes have their roots in theoretical biology, and are nowadays widely used to describe the dynamics of both evolutionary and local search algorithms (where they are referred to as search landscapes). Key to this concept are an arrangement of possible solutions (or genotypes) in an abstract space that describes how solutions can be reached from one another, and a fitness or objective function assigning a quality value (height) to all solutions, forming a surface. The intuitive notion of *ruggedness* is related to the difficulty of optimising on a given landscape. It depends on both the fitness function and the geometry of the search space, which is induced by the search operators. Additionally, studies of molecular evolution [24] have shown that *neutrality*, that is, the occurrence of adjacent configurations with the same fitness, can also play a dominating role in evolutionary dynamics.

The goal of fitness landscape analysis is to provide a detailed understanding of the geometric features of landscapes and how they relate to search dynamics. However, mountain ranges, valleys, basins, peaks, plains and ridges in multi-dimensional combinatorial objects may look quite different from our 3D experience [32]. A wealth of metrics and techniques have been proposed to characterise fitness landscapes [22], but most of them study the local structure of search spaces. Local Optima Networks (LONs) [25, 41], help us to study instead their global structure. This graph-based model provides fundamental new insight into the structural organisation and the connectivity pattern of a search space with given move operators. Most importantly, it allows us to visualise realistic search spaces in ways not previously possible and brings a whole new set of network metrics for characterising them.

Genetic improvement (GI) uses automated search to find improved versions of existing software [29]. The goal is to automatically improve the behaviour of a software system with respect to some desired criteria using heuristic search. The criteria for improvement can be either non-functional properties of the system, such as execution time or power consumption; or functional properties such as repairing defects (also called Automatic Program Repair [45]). Despite being a recent topic, GI has received awards and notoriety within both Software Engineering and Evolutionary Computation [29]. Several challenges can be identified when trying to automatically improve software [17], among them is to understand the structure of program search spaces. Within GI, a program is mutated in an attempt to improve some desired property. For example, in a bug-fixing scenario, the fitness of a program is measured by the number of failed test cases from a given test suite. Neutrality is relevant as it is related to the proportion and distribution of test-equivalent mutants. Traditionally, the space of program mutants has been thought to be disjoint and fragile, with few good programs. However, recent work [18, 19, 30, 46] suggests that many changes do not impact the fitness of the mutants. This may indicate that either the programs are quite robust, or the test suite does not provide enough coverage.

Visualisation has been recognised as an important tool for understanding evolutionary algorithms. Insightful reviews of existing techniques can be found in [10, 21]; most approaches visualise either search trajectories (evolutionary histories), the structure of individuals, the ancestry of individuals, or the composition of populations. Concerning the visualisation of fitness landscapes, the recent work by Volke et al. [42, 43] collects data from steepest descent walks and computes a set of distance measures, which are thereafter visualised as a set of scatter plots, bar charts, and 2D projection maps. The mapping is done using a spring force model such that the Euclidean distances between the points in the plane approximate the distances within the search landscape. Another direction of research uses the notion of barrier trees or disconnectivity graphs [44] from theoretical biology for visualising the search landscape. These studies mostly deal with small examples. Flamm et al. [8] extended barrier trees so that they can be constructed for highly degenerate problems (i.e., landscapes with neutrality). They present empirical results for binary strings of up to length 10. Hallam and Prügel-Bennett [9] constructed barrier trees for MAX-SAT problems with up to 40 variables using branch-and-bound to find only the best local optima in the space.

It is not the intention of this paper to propose a fully fledged standalone solution for visualising search landscapes and networks. Rather, its purpose is to highlight that existing visualisation techniques can be added to the researcher’s or the practitioner’s toolkit when analysing and communicating results of a local optima network analysis. Furthermore, applying those techniques to a number of genetic improvement scenarios demonstrates the applicability of the techniques to a broader range of problems beyond traditional combinatorial optimisation benchmark problems such as the Travelling Salesman Problem.

In this paper, we propose using the t-distributed stochastic neighbour embedding, or t-SNE algorithm [36], as the dimensionality reduction technique to generate layouts of large local optima networks datasets. The t-SNE algorithm, while very popular in machine learning, has received very little attention in an optimisation context. It has previously been used to visualise the search dynamics in Particle Swarm Optimisation on continuous functions [6] where it is briefly compared to Principle Components Analysis. Other dimensionality reduction techniques such as Sammon mapping [7, 13] and isometric mapping [15] have been proposed for visualising evolutionary algorithm runs.

This article extends our recent work on modelling genetic improvement landscapes as local optima networks [38] and explicitly focuses on the visualisation aspects. The main contributions are:

1. Combining local optima networks and dimensionality reduction through the t-SNE algorithm to visualise the global structure of search landscapes.
2. An investigation of the biases of the visualisation in terms of (1) how different samples compare visually to each other and (2) how different sampling intensity can bias the proposed visualisation by comparing the visualisation of a large set of solutions with the visualisations of a number of its subsets.
3. An analysis illustrating how the visualisations can be used to compare different landscape samples generated by two different algorithms (an ILS and a GA).

The paper is structured as follows. Section 2 describes fitness landscapes and local optima networks. Section 3 presents the programs used in our case study and which of their features are mapped onto the landscape. Section 4 describes the algorithm used for genetic improvement in this paper and, specifically, the Iterated Local Search and hybrid genetic algorithm used. Section 5 discusses some considerations when visualising large graphs and local optima networks in particular. Section 6 presents the visualisation results and Sect. 7 is the conclusion.

## Search landscapes

**Fig. 1:**
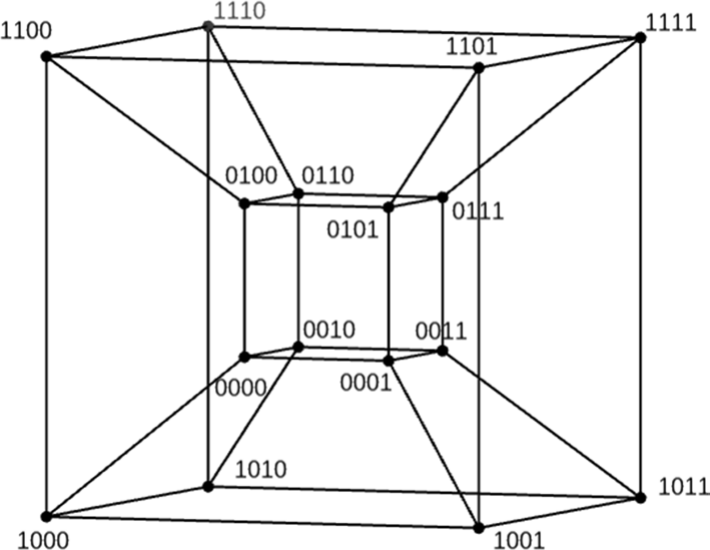
Search space of bit strings of length 4.

In practice, the two or three-dimensional visual interpretation of the *landscape* metaphor is not directly possible for high-dimensional search spaces and some dimensionality reduction is required. Indeed, while the search space of a four-dimensional binary problem (only 16 solutions) under the bit flip neighbourhood can be represented by a tesseract or hypercube (Fig. 1), conveying the additional fitness dimension of the landscape is non-trivial if one wants to maintain the intuitiveness of the metaphor. Naturally, colour or some other property may be used to illustrate fitness. Nevertheless, there are formal mathematical objects that can be associated to the landscape metaphor to allow for its analysis and manipulation. The rest of this section describes fitness landscapes and local optima networks in more detail.

### Fitness landscapes

Fitness landscapes are a commonly-used metaphor to describe the dynamics of evolutionary and local search heuristics. The search space can be regarded as a spatial structure with height representing fitness, forming a surface that can vary from smooth to rugged, and that can contains ridges and plateaus. Formally [32], a landscape is a triplet (*S*, *N*, *f*) where

- *S* is a set of potential solutions, i.e. a search space,
- $N~:~S~\longrightarrow ~\wp (S)$, the neighbourhood structure, is a function that assigns to every $s \in S$ a set of neighbours *N*(*s*) ($\wp (S)$ is the power set of *S*), and
- $f : S \longrightarrow {\mathbb {R}}$ is a fitness function that can be pictured as the *height* of the corresponding solutions.

Within the landscape structure, different topological features such as local optima, basins of attraction and plateaus may appear. These are known to have an impact on the search process.

#### **Definition 1**

(*Local optimum*) A local optimum is a solution $s^{*} \in S$ such that $\forall s \in N(s^{*})$, $f(s^{*}) \le f(s)$. Notice that the inequality is not strict, in order to allow the treatment of the neutral landscape case. The definition provided here is for a local minimum, without loss of generality.

#### **Definition 2**

(*Basin of attraction*) The basin of attraction of a local optimum $s^{*}_i \in S$, for some hillclimbing operator *h*, is the set $b_i = \{s \in S | h(s) = s^{*}_i \}$.

#### **Definition 3**

(*Plateau*) A plateau is a set of connected solutions with the same fitness value. Two vertices in a plateau are connected if they are neighbours with the same fitness.

### Local optima networks

Local optima are important features of fitness landscapes as they can be seen as obstacles to the progress of heuristic search. Local optima networks (LONs) [25] model the global structure of landscapes as graphs where nodes are local optima and edges represent possible transitions among them with a given search operator. In order to model GI fitness landscapes with local optima networks, we adapted the model with escape edges [40]. To construct these networks, we need to define their nodes and edges. The definitions are related to the search operators used, specifically, the local search (hill-climbing) heuristic to determine the local optima and *escape* operator to transit among them. In our study, the hill-climbing heuristic is a best-improvement approach based on the 1-move operator, and the escape operator is also given by a single application of 1-move. This is possible as, given the problem encoding, a single 1-move provides enough variability to escape from a local optimum basin of attraction.

#### **Definition 4**

(*Nodes*) Nodes in a LON are *local optima*. In genetic improvement landscapes, a local optimum is a minimum when considering the number of failed test cases. The set of local optima, which corresponds to the set of nodes in the network model, is denoted by *L*.

Since the whole set of local optima cannot be determined in realistic search spaces, such as those considered here, a process of sampling is required to estimate *L*.

#### **Definition 5**

(*Edges*) Edges are directed and based on some operator(s) that allow for the transition between two local optima. The type of edge will differ based on the type algorithm that is considered. The set of escape edges is denoted by *E*.

The type of edge is dependent on the algorithm used to sample the landscape. While escape edges are natural edges for ILS algorithms, other edge types may be used such as crossover and mutation edges in the case of an evolutionary algorithm, as done by [39].

In this paper, we consider *escape* and *crossover* edges. There is an *escape* edge from local optimum *x* to local optimum *y*, if *y* can be obtained after applying a 1-move random mutation to *y* followed by a best-improvement hill-climb across the 1-move neighbourhood. There is a pair of *crossover* edges from local optima $x_1$ and $x_2$ to local optimum *y*, if applying a uniform crossover on $x_1$ and $x_2$, followed by a best-improvement hill-climb across the 1-move neighbourhood produces *y*.

#### **Definition 6**

(*Local optima network (LON)*) This is the $LON = (L,E)$ graph where nodes are the local optima *L*, and edges *E* are the escape edges.

## Program search space test bench

To study the landscape of program search spaces, we start from known bug-free programs and introduce random mutations. Starting from these mutants, we try to recover the original bug-free programs or any version that passes all the test cases in the test suite.

We assume that programmers usually create close-to-being-correct programs, i.e., they make minor mistakes such as typos or small logic errors but not major mistakes where the program logic is completely flawed. This is known as the *competent programmer hypothesis* [4].

We examine two C programs, described in Sect. 3.1. For the sake of simplicity, in this paper we first consider mutations on comparison operators ($\texttt {<},~\texttt {<=},~\texttt {==},~\texttt {!=},~\texttt {>=},~\texttt {>}$), as done by [16], and then add mutations of Boolean operators with two operands (&&, ||) to compare and contrast landscapes across different neighbourhood structures.

**Fig. 2:**
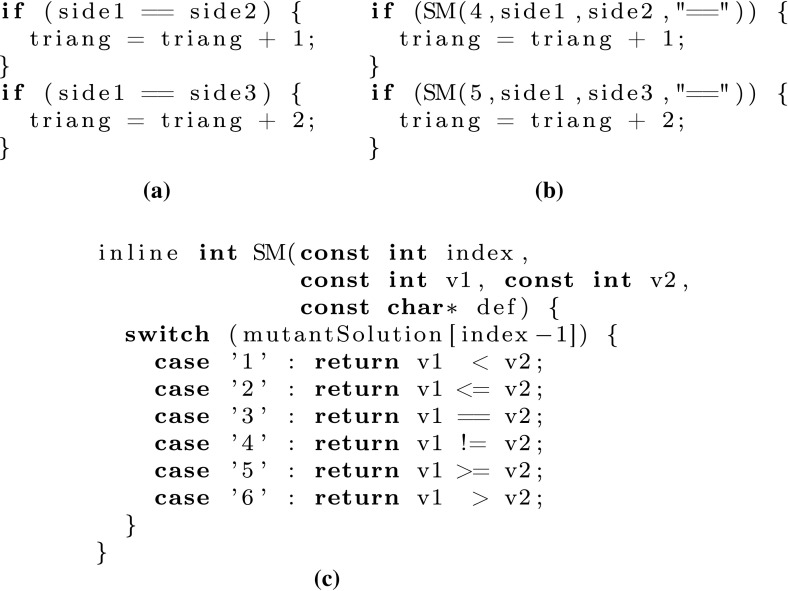
Code snippets showing an example of super-mutant transformation where the two equality operators are the fourth and fifth operators in the code. **a** Original code snippet, **b** super-mutant code snippet, **c** SM function definition

To bypass the generation of new code requiring the recompilation of each new mutant, we use a *super-mutant* program that contains all the possible mutations under consideration [34]. These mutations can then be turned on and off as desired. In our implementation, each operator is transformed into a function call with four arguments: an operator id, its two operands, and a cosmetic final argument string that describes the original operator. This is mostly useful for a fast evaluation of the fitness function. An example of the transformation is shown in Fig. 2.

The transformation is performed using the LibTooling library of Clang–LLVM to parse the programs, build the abstract syntax trees, and rewrite the required nodes. Some additional manual steps are required to build our test harness, as described in Sect. 3.1.

In the programs we examine, no two operators are ever part of the same expression when only the comparison operators are considered. When both comparison and Boolean operators are considered, then operator precedence is enforced and left associativity is used for operators of similar precedence when the super-mutant is generated.

In our study, a potential solution is encoded as a vector of integers of length *l*, where $l = c + b$ corresponds to the number of comparison operators (*c*) and Boolean operators (*b*) in the program under consideration (Table 1). There are 6 possible comparison operators and 2 Boolean operators. Therefore, the size of the search space is $|S| = 6^c \times 2^b$. The neighbourhood structure, *N*, is given by the simplest possible move operator in this landscape, namely, the value of a single position in a solution is changed: to one of the 5 alternatives if it is a comparison operator, or the opposite Boolean operator. Let us call this operator *1-move*. The size of the neighbourhood induced by 1-move on the given representation is $5\times c + b$. The fitness function *f* is given by the number of test cases failed by the program, which is to be minimised.

### Benchmark programs

We use two C programs: the triangle program and the TCAS program. Their characteristics are summarised in Table 1.

triangle.c: The triangle program is a small program that takes the lengths of the three sides of a triangle and determines if it is scalene, isosceles, equilateral, or not a triangle. We use a simplified version [16], which has been translated into C from the original Fortran version by DeMillo et al. [4].

tcas.c: The TCAS, or Traffic Collision Avoidance System, program controls the altitude of an aircraft depending on a number of input parameters. We use version 2.0 from the SIR repository [5].^1^ For the sake of simplicity, we do not consider the test cases that do not have all 12 input parameters. This effectively reduces the number of test cases from 1608 to 1578. Array indices are not checked in the original program. We introduce a check to accept valid indices and generate an arbitrary output value for invalid indices. This prevents the program from crashing and improves the efficiency of the sampling process, which is described in the next subsection.

**Table 1:** Characteristics of benchmark programs

Program	triangle.c	tcas.c
Lines of code	40	135
No. comparison operators	17	14
No. Boolean operators	7	16
No. input parameters	3	12
No. output values	1	1
No. test cases used (original)	14 (14)	1578 (1608)

## Genetic improvement sampling procedures

A full enumeration of the search space, or even of the local optima, for the two programs is unmanageable. Therefore a sample of high-quality local optima in the search space is generated. Since we only consider mutations of comparison and Boolean operators, a simple representation for a solution is a vector of integers. Consequently, any metaheuristic could be used to explore the search space—provided that it also generates local optima for the LONs. Here we consider Iterated Local Search and a genetic algorithm hybridised with local search.

### Iterated local search

Iterated Local Search, or ILS (Algorithm 1), starts from a locally-optimal solution and then alternates between a random mutation and a best-improvement hill-climber. The termination criterion is a fixed number of iterations. At each step, only non-worsening local minima are accepted. The fitness, or objective value, of a solution is the number of test cases that it fails. Both the hill-climber and the mutation consider the first degree or 1-move neighbourhood, i.e., neighbouring solutions only differ by a single element. To build the networks, the ILS is run 1000 times and the stopping criterion for each run is 10000 iterations.
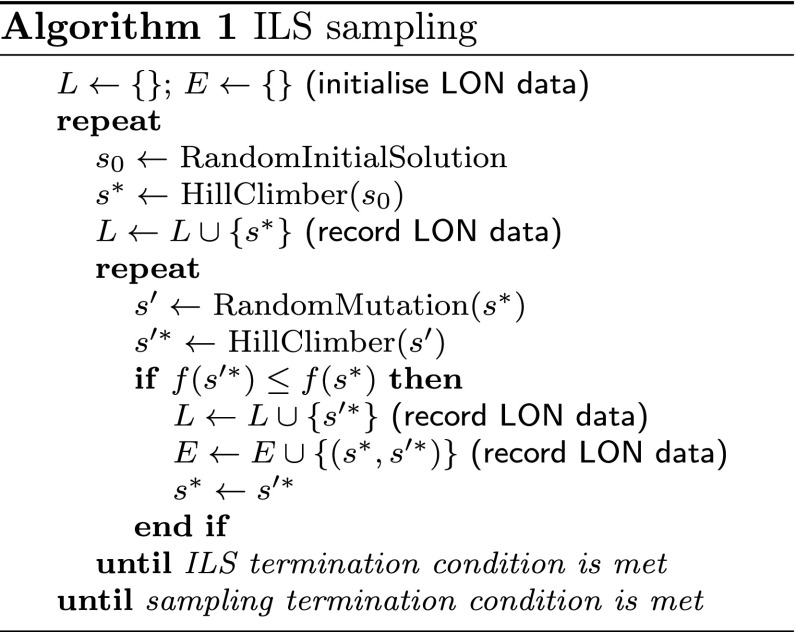


### Genetic algorithm with local search

The genetic algorithm with local search, or Hybrid GA (Algorithm 2), is simple. It applies uniform crossover to 2 parents in order to produce 2 offspring. This is immediately followed by a best improvement hillclimb on the 1-move neighbourhood to obtain a local optimum. The Hybrid GA does not employ mutation. Binary tournament selection is used. The crossover is applied with a probability of 0.5. Generational replacement is employed. The algorithm uses a population of 400 solutions for 50 generations. The networks are built by running the Hybrid GA 1000 times.

Since crossover is applied only half the time, there should potentially be about 200 new solutions created per generation, or 10000 solutions across 50 generations. This is meant to roughly match the computational effort for the same number of iterations in the ILS.
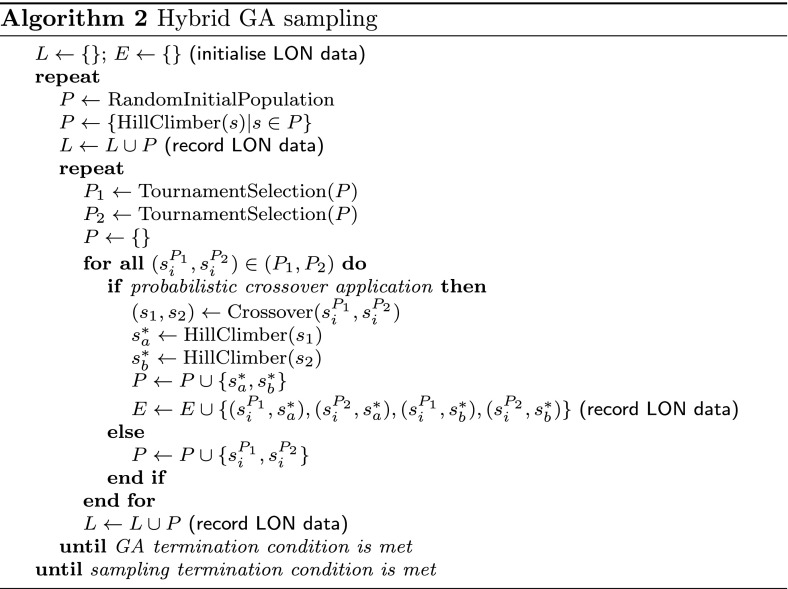


## Visualising local optima networks

Since they are network objects, it is natural to wish to visualise LONs, as one would for social networks or protein interaction networks. In particular, while it is easy to compute network metrics that describe certain properties, visual presentation, exploration and analysis [12] can allow for the communication of a richer set of information and reveal patterns or incongruities. In the case of LONs, this may be how solutions of similar fitness are grouped together or what are the connectivity patterns of suboptimal solutions to global optima or to other local optima, thus filling some of the analytic gaps [1] of a purely metric-driven approach. These may indicate how well—or not—the search space is being explored by some algorithm or how two algorithms explore the space differently.

Local optima networks can be visualised as graphs in two or three dimensions. Although they are a compressed representation of fitness landscapes, they are still usually too large for allowing a clear representation of non-trivial landscapes due to computational limitations and an overload of data, for instance when the graph becomes a complex hairball.

Nodes and edges in a network can be formatted in different ways in order to highlight different properties. Usually this is achieved by changing their colour, their width (edges), or size (nodes). Moreover, different techniques can be applied in order to highlight topological properties of a large network. This can be done, for instance, by limiting the amount of information, or objects, displayed. Another way is to choose appropriate layout techniques. These issues are discussed further in this section.

### Handling large networks

Since our objective is to visualise the global structure of fitness landscapes, we want to be able to display a large number of local optima and the connections between them. Large, here, means at least of the order of a thousand but usually of the order of tens of thousands, which can still be represented relatively clearly both on screen and on paper. The sampling algorithms do not exhibit such limitations and it is computationally feasible to generate many more local optima—in the order of millions and above—from a complex landscape. Different techniques can be used for such large datasets.

Given a large dataset of local optima, selecting a representative subset can effectively reduce the number of points. For example, a threshold on the nodes’ fitness can be used in order to only observe the part of the network that is closer to the global optimum, which is arguably one of the most interesting portions. As we will see, the landscapes considered here exhibit neutrality and usually contain a large number of global optima. Using a threshold on fitness in this context is therefore not ideal since the resulting visualisation will display few large flat plateaus, and fail to display the general connectivity patterns. Having a threshold on fitness, however, might still be useful when investigating the connectivity within plateaus of very good fitness, or for non-neutral landscapes in other contexts.

Another way of selecting a representative subset of local optima is according to the exploration parameters of the algorithm generating the local optima dataset. This is the approach we follow here. We restrict the nodes and edges displayed to those produced by a portion of the runs and iterations or generations per run of the data collecting algorithm. This is especially adequate in the context of this article, as for the selected instances, there are always a number of runs that converge early to a global optimum.

We also want to avoid occlusion of adjacent nodes and overlapping edges. This can be achieved with appropriate layout algorithms (Sect. 5.2) or by only rendering a subset of the elements. Since, by construction, there are no or few isolated nodes in our networks we can dispense from displaying the nodes and choose to display only the edges thus focusing on the connectivity patterns. This works especially well in 3D. Alternatively, edges can be hidden and only nodes displayed to avoid edge hairball scenarios (in 2D).

### Layout algorithms

Layout algorithms generate coordinates for each node of the network. In this paper, we consider 2D layouts, which can be seen as providing a bird’s eye view of the landscape. These 2D layouts can be augmented by fitness as a third dimension. The associated 3D representation can then be interpreted in an intuitive manner as it evokes to some extent the mental picture of the landscape metaphor.

Our previous work has exclusively focused on force-directed layouts to visualise LONs. A contribution of this paper is to use dimensionality reduction as a layout technique to plot the nodes, such that their distribution conserves the similarity of optima in genotypic space. Both approaches are briefly described hereafter.

### Force-directed layouts

Force-directed layout algorithms [14], also known as spring embedders, rely on the structure of the graph to compute its layout and do not consider domain-specific knowledge. Such layouts tend to be aesthetically pleasing: exhibiting symmetry and minimising edge crossing for planar graphs.

Vertex attraction and edge repulsion forces are assigned to the set of vertices and edges. These can be based on spring-like attraction forces between nodes, for instance based on Hooke’s Law, while simultaneously modelling repulsive forces to separate pairs of nodes, like the force between electrically charged particles based on Coulomb’s law.

In this paper we consider the *Dr.L* algorithm [23]. This is a multilevel force-directed algorithm based on simulated annealing. At each level, the layout is clustered to produce a coarsened graph with fewer nodes. The smallest graph generated is used as a basis for drawing the original graph by refining the series of coarsened graphs that were produced. In this manner, Dr.L is able to efficiently handle large-scale graphs.

### Dimensionality reduction

Force-directed layouts are based solely on the connectivity of the graph and do not consider any property of nodes. This naturally influences the attributes of the visualised landscape, with connectivity between vertices influencing how close the vertices are together.

In order to visualise some aspect of similarity between solutions, additional problem-specific information is required. Furthermore, the solutions are originally in a high-dimensional space and need to be represented in two or three dimensions by using dimensionality reduction techniques. The aim of dimensionality reduction is to preserve as much of the significant structure of the high-dimensional data as possible in the low-dimensional map. A number of different methods have been proposed and they differ in terms of the types of structures they preserve. In this paper we mainly consider the t-SNE [36] technique because it is one of the few non-linear techniques which are scalable enough to efficiently compute layouts for tens of thousands of points. t-SNE stands for t-distributed stochastic neighbour embedding and has become fairly popular, especially for machine learning datasets. It generally has the nice property of reflecting both the local and global structure of the data.

We also briefly consider Principal Components Analysis (PCA) since it is widely used for dimensionality reduction and can also scale well. PCA is a linear technique that aims at keeping low-dimensional representations of dissimilar points far away. Its downside is that it cannot be used on categorical data—types of comparison and Boolean operators in our context. The counterpart to PCA for such data is Multiple Correspondence Analysis (MCA). It can be viewed as applying PCA to the complete disjunctive table, i.e., the binary table where columns are the variable-category pairs of the original data.

**Fig. 3:**
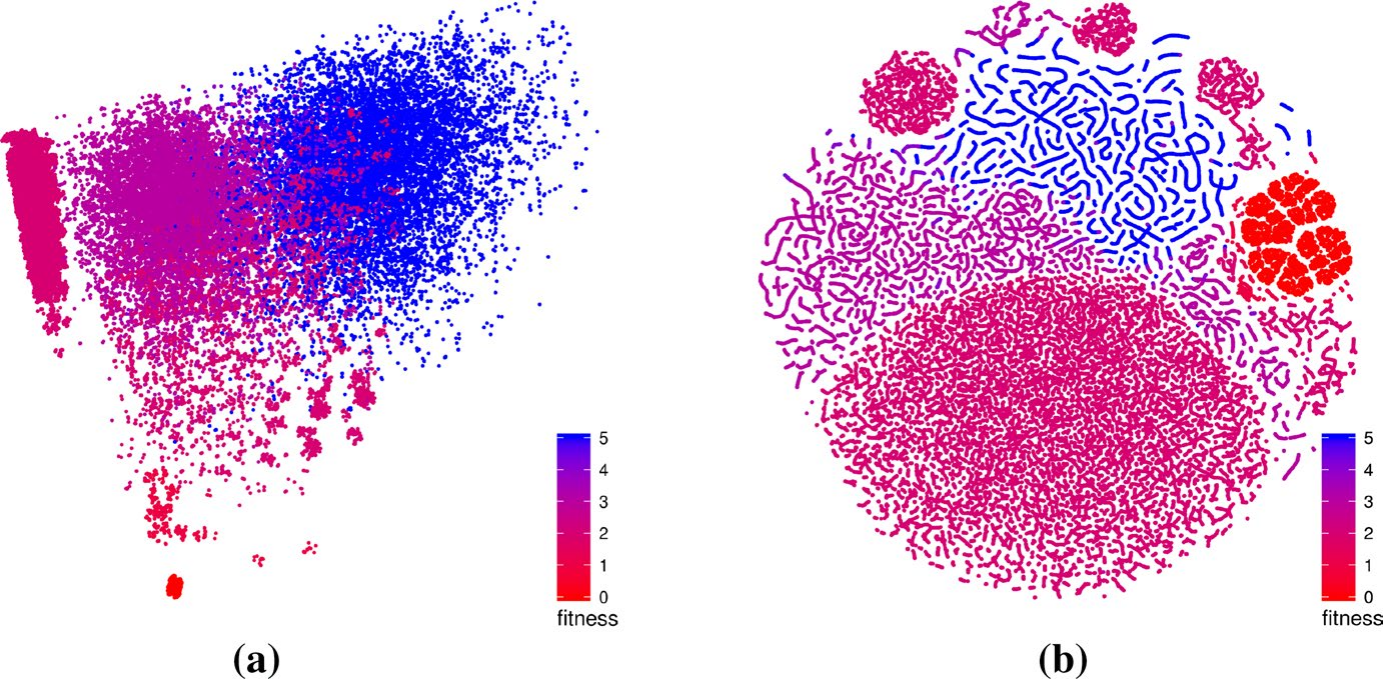
Comparison of MCA and t-SNE layouts for local optima sampled through ILS. Solutions are coloured according to their fitness. The MCA layout reveals some structure but the t-SNE layout highlights more patterns in the data. **a** MCA layout, **b** t-SNE layout (Color figure online)

Figure 3 compares an MCA layout and a t-SNE layout for the same original data. While MCA is able to show some structure in the data—especially showing solutions at the same fitness level being in the vicinity to each other—t-SNE is able to extract and display additional structure—for instance revealing that very similar solutions form worm-like artefacts, that are in fact subsections of the search trajectories, or allocating space to very similar solutions to highlight local structures (at fitness level 0 for example). t-SNE is described in some further detail next.

Many non-linear dimensionality reduction techniques have been proposed and several of them are reviewed by Lee and Verleysen [20]. These non-linear techniques aim to preserve the local structure of the data but have not been very successful in preserving both the local and global structure of the data in a single map.

t-SNE minimises the divergence between the distribution that measures pairwise similarities of high-dimensional points and the distribution that measures pairwise similarities of the corresponding low-dimensional points. The latter distribution is computed as a normalised Student-t kernel with a single degree of freedom which explains the name of the algorithm. Since the normalised Student-t kernel has heavy tails, this allows for dissimilar points to be modelled by low-dimensional counterparts that are also far apart. This creates more space to accurately model small pairwise distances, or local structure, in the low dimensional space. The Kullback-Leibler divergence between the two distributions is minimised. The objective function is non-convex and is minimised by gradient descent. Therefore, images generated from the same set of points but not using the same random seed may be different. In practice, in our context of tens of thousands of points, the major visible differences are mirroring and rotation of the points. Indeed, as discussed and shown in Sect. 6.3, even different sets of points produce very similar images, as long as they have been sampled in the same way.

The computational complexity of the Barnes-Hut version of t-SNE [35] is only ${\mathcal {O}}(n\log {}n)$, whereas most other methods are usually at least quadratic in their complexity [37]. It can thus handle a large number of data points.

## Visualisations

This section starts by looking at some technical implementation choices and then discusses the results and visualisations.

### Implementation choices

The implementation is not a stand-alone program but a set of scripts, mainly written in the R programming language but also in Python, in order to be easily integrated within current workflows for local optima network analysis. The implementation choices allow for programmatically generating and manipulating large networks and their static visualisations at the expense of more interactive approaches, for instance based on GUI network visualisation solutions such as Cytoscape [31] or Gephi [2].

The networks are built with the igraph library [3] in R. All the layouts are generated in two dimensions using either the Dr.L force-directed layout algorithm, as implemented in igraph, or the t-SNE algorithm, as implemented in the scikit-learn package in Python (and with the default parameters). This choice was made because scikit-learn provides the ability to easily change the dissimilarity metric used in the algorithm: the Hamming distance in our case instead of the Euclidean distance. The 3D networks are created by adding fitness as a third dimension, or height, to the 2D layouts. Rendering the images is carried out in R using the ggplot2 package to create the scatterplots and the rgl package to create OpenGL 3D output.

### Discussion

The following encompasses a number of different aspects of our results. We first look at the characteristics of the visualisations (Sect. 6.2.1) and then discuss how they relate to the network objects when considering the ILS samples (Sect. 6.2.2). This is followed by examining the influence of different subsamples on the visualisations (Sect. 6.3) and a comparison between the ILS and Hybrid GA samples (Sect. 6.4).

**Fig. 4:**
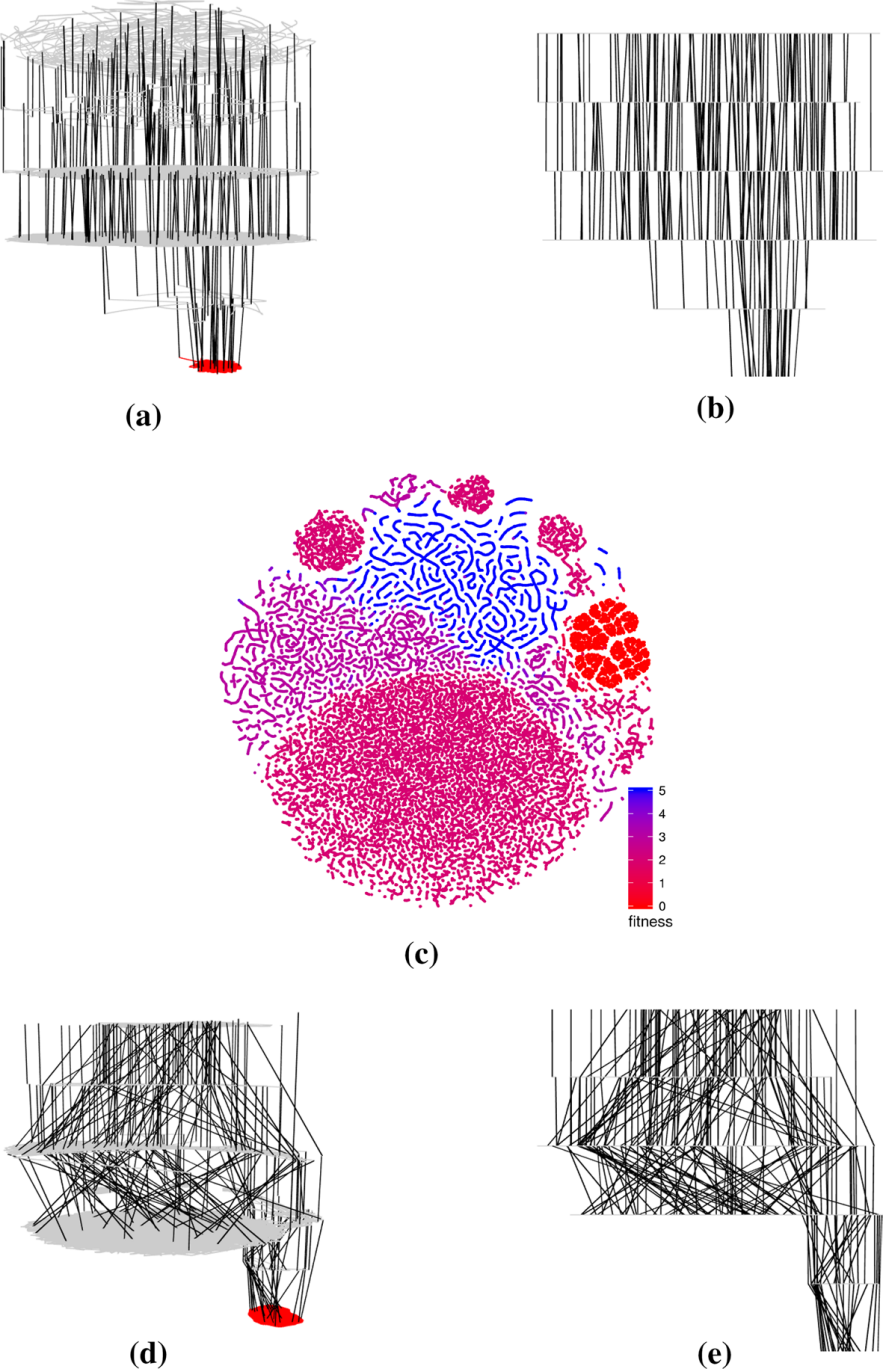
Triangle program, comparison operators only (success rate: 87.1%)—subsampled LON of 100 runs of 1000 iterations. In the networks, there are multiple paths that lead to global optima (red). The t-SNE scatterplot shows that solutions of similar fitness are grouped together and global optima occupy only a small section of the sampled search space. **a** 3D view of force-directed layout, **b** profile view of force-directed layout, **c** t-SNE layout, **d** 3D view of t-SNE layout, **e** profile view of t-SNE layout (Color figure online)

Figures 4 (triangle program with comparison operators only), 5 (triangle program with comparison and Boolean operators), 6 (tcas program with comparison operators only), 7 (tcas program with comparison and Boolean operators) show visualisations of the LONs. The figures show a subsample of the first 100 runs of the original sample and the first 1000 iterations of each of these. The scatter plots are the layouts generated by the t-SNE algorithm. Nodes in the 3D images are not displayed to minimise occlusion. However, the edges by themselves provide insight into the nature of the landscapes. Edges between global optima are painted red and edges between local optima of equal fitness are painted grey. Edges between local optima with different fitness are painted black.

**Fig. 5:**
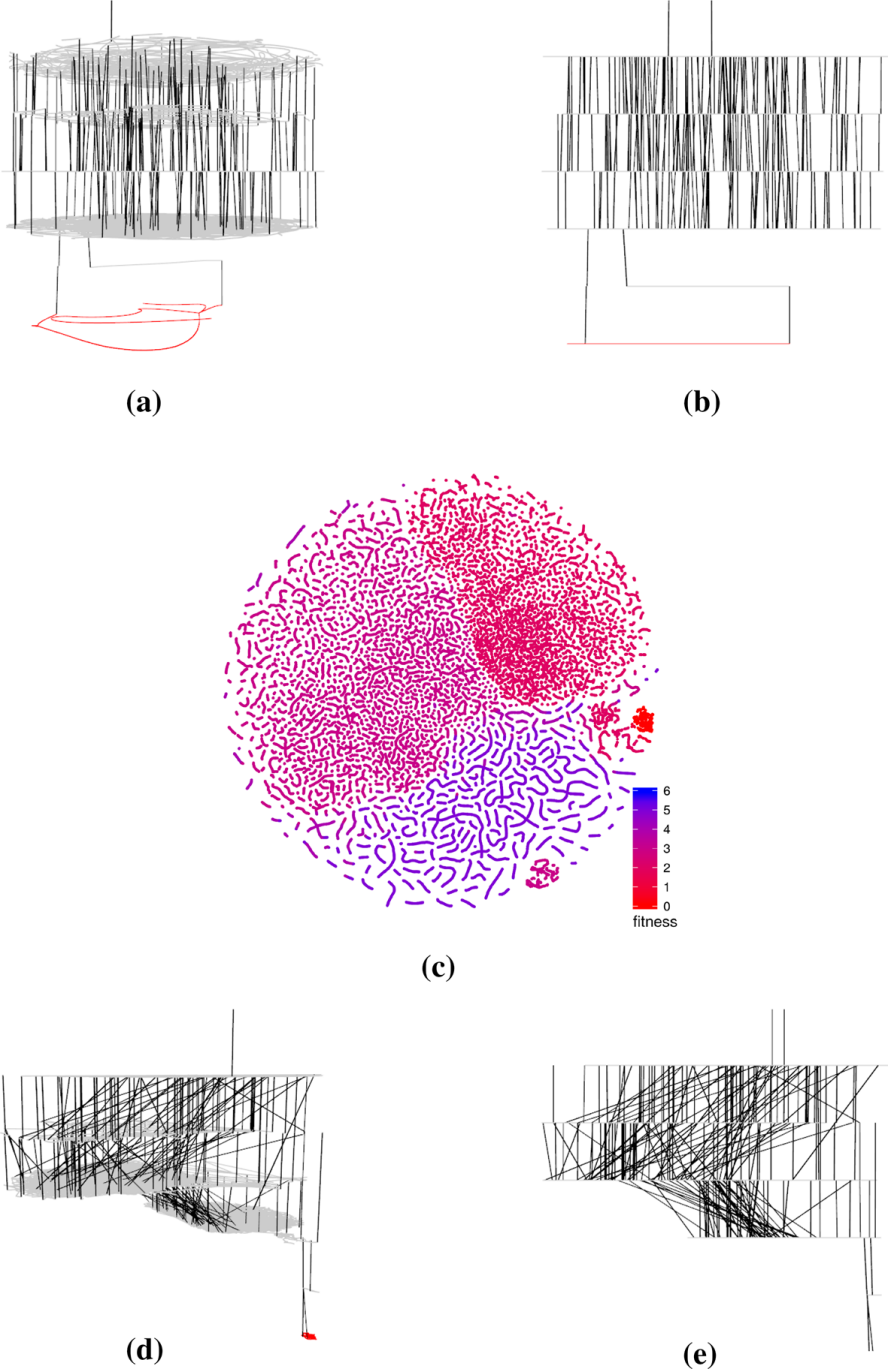
Triangle program, comparison and Boolean operators (success rate: 31.2%)—subsampled LON of 100 runs of 1000 iterations. In the networks, there are multiple paths between the fitness levels that are not the best but few that lead to global optima (red). The t-SNE scatterplot shows that solutions of similar fitness are grouped together and global optima occupy only a comparatively small section of the sampled search space. **a** 3D view of force-directed layout, **b** profile view of force-directed layout, **c** t-SNE layout, **d** 3D view of t-SNE layout, **e** profile view of t-SNE layout (Color figure online)

**Fig. 6:**
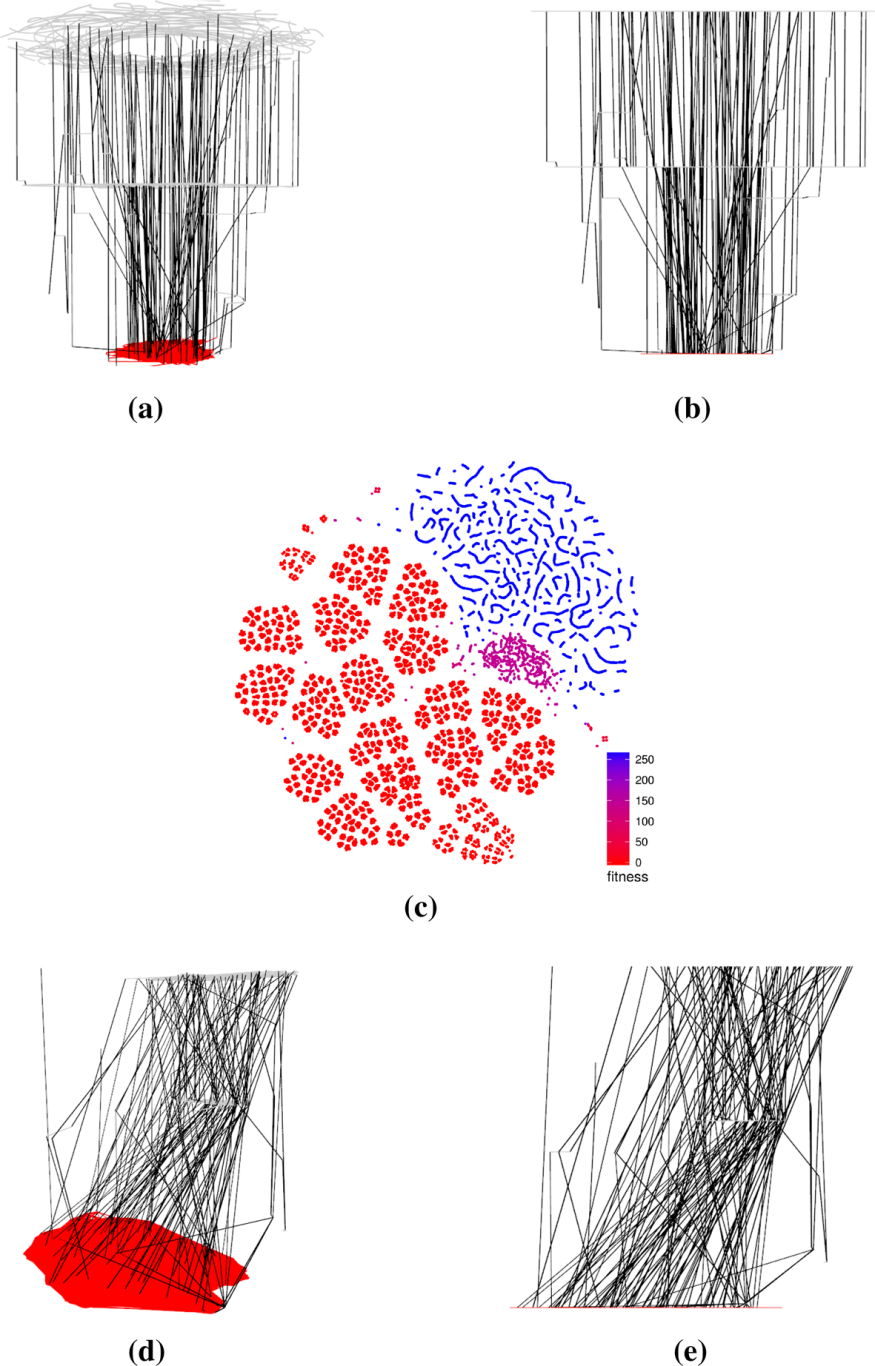
Tcas program, comparison operators only (success rate: 94.4%)—subsampled LON of 100 runs of 1000 iterations. In the networks, there are multiple paths that lead to global optima (red) and these paths traverse the different fitness levels fairly directly, save for two fitness levels (at the top and the middle) were multiple trajectories wander across meta-plateaus. The t-SNE scatterplot shows global optima occupy a comparatively large portion of the sampled search space. These global optima are well connected together in the networks. **a** 3D view of force-directed layout, **b** profile view of force-directed layout, **c** t-SNE layout, **d** 3D view of t-SNE layout, **e** profile view of t-SNE layout (Color figure online)

**Fig. 7:**
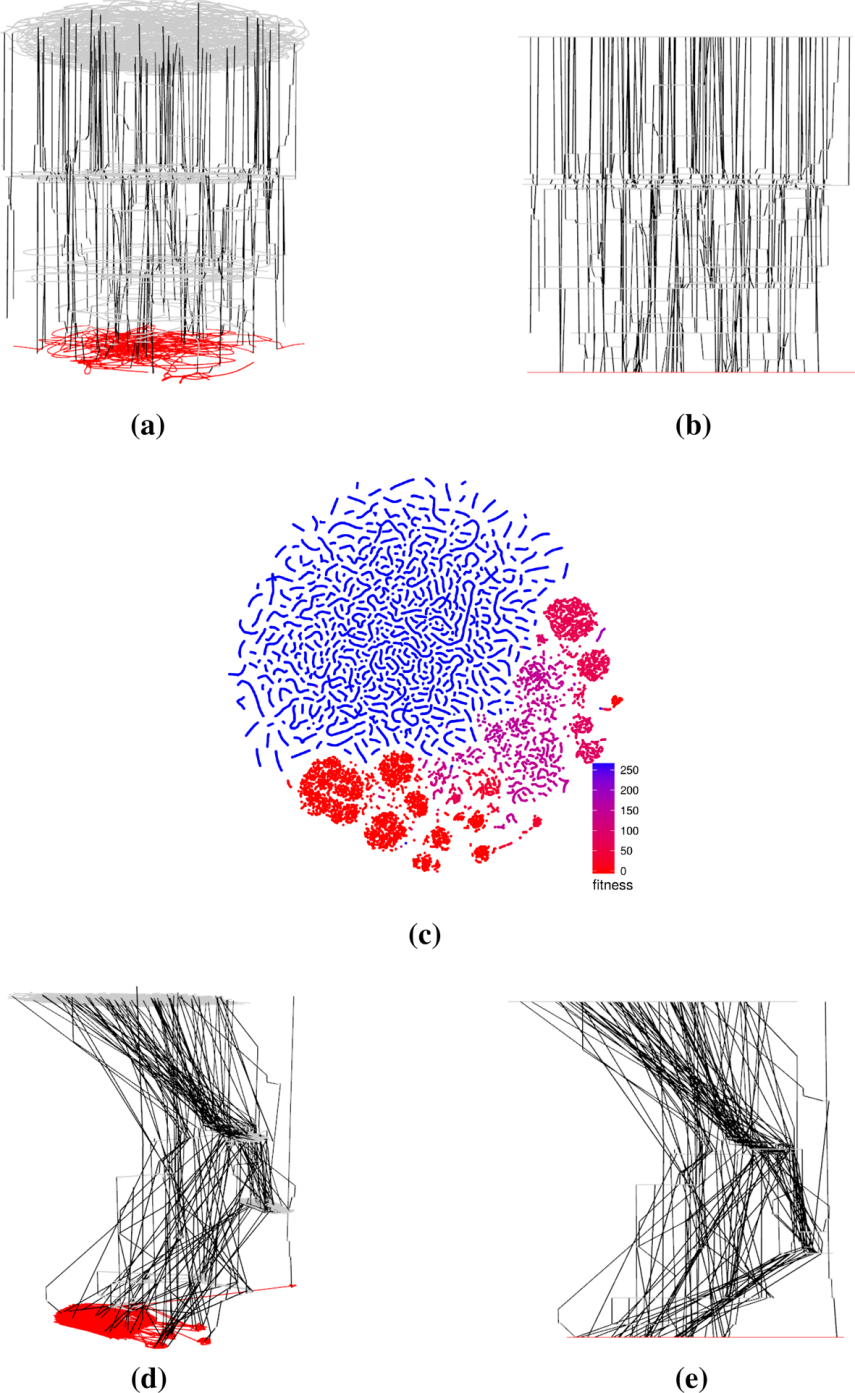
Tcas program, comparison and Boolean operators (success rate: 98.4%)—subsampled LON of 100 runs of 1000 iterations. In the networks, there are multiple paths that lead to global optima (red). These paths are fairly direct but exhibit some “staircase” patterns where the trajectories travel along the same fitness for a short while. The t-SNE scatterplot shows that solutions of similar fitness are grouped together and global optima occupy a subset of the sampled search space. **a** 3D view of force-directed layout, **b** profile view of force-directed layout, **c** t-SNE layout, **d** 3D view of t-SNE layout, **f** profile view of t-SNE layout (Color figure online)

#### Characteristics of the visualisations

The force-directed layouts (subfigures (a) and (b) in Figs. 4, 5, 6, 7) display fairly symmetric layouts which are characteristic of the force-directed algorithms. Since no information about the solutions is taken into account when computing the layout, the different plateaus usually appear more-or-less on top of each other. Furthermore, edges linking different plateaus are generally vertical. This may influence the viewer into thinking, wrongly, that solutions at each end of those edges are always genotypically close.

On the other hand, the t-SNE algorithm uses the similarity between the solutions to infer meaningful coordinates (subfigure (c) in Figs. 4, 5, 6, 7). In each of the scatter plots, clear local and global structures emerge. Locally, there are a number of worm-like artefacts which are composed of series of consecutive and very similar points generated by the sampling process. This happens because the sampling process generates monotonic sequences of solutions and accepts non-worsening moves (solutions of same fitness). In addition, the first degree—or 1-move—neighbourhood is considered for both mutation and hillclimbing, producing generally small local steps in which two consecutive solutions are very similar to each other. Since the t-SNE algorithm relies on the similarity between points to determine their position relative to each other—and the measured Hamming distance similarity corresponds to as many 1-moves—this translates into these worm-like structures.

More globally, solutions with the same fitness seem to cluster together, indicating plateaus of solutions that occupy different areas of the search space.

When fitness is added as a third dimension to the t-SNE layouts (subfigures (d) and (e) in Figs. 4, 5, 6, 7), we can observe a more accurate picture of the sampled search space than when the force-directed layout was used. In particular, it is visible that moving from one plateau to another often involves moving to an altogether different part of the landscape. This naturally creates a large number of crossing edges which may be considered less aesthetic but which convey more information. There would, however, be a point, as the number of displayed edges increases, where the visualisation would become an uninformative hairball. One way around this, but which is beyond the scope of this paper, may be *edge bundling* [11], where related edges are routed along similar paths, thus minimising edge clutter.

#### Relating the visualisations to the network objects

Table 2 reports the main characteristics of the LON graphs extracted from the benchmark problems described in Sect. 3.1. The sampling procedure yielded, in all cases, graph sizes in the order of one million edges, which are non-deteriorating transitions between local minima. The actual number of distinct local minima visited during the search, that is, the number of nodes in the graph, is also in the order of one million for triangle.c, and one order of magnitude less in the case of tcas.c. In particular, allowing mutations to both comparisons operators and Boolean operators, increases the size of the search space and the number of local minima.

**Table 2:** Network characteristics and ILS performance

Program	triangle.c		tcas.c	
Variant	c	c $+$ b	c	c $+$ b
No. of nodes	2,432,263	4,063,871	85,621	504,866
No. of edges	2,758,358	4,670,188	641,034	1,436,281
No. of global optima	9216	53,897	22,824	114,412
Network density	${{4.7}} \times 10^-{7}$	${{2.8}} \times 10^{-{7}}$	${{8.7}} \times 10^{-{5}}$	${{5.6}} \times 10^{-{6}}$
Clustering coefficient	${{2.4}} \times 10^{-3}$	${{2.4}} \times 10^{-3}$	${{4.4}} \times 10^{-2}$	${{1.6}} \times 10^{-2}$
Neutral degree	99.8%	99.9%	99.6%	99.6%
No. of connected components	3	3	2	12
Relative size of largest conn. comp.	92.6%	99.8%	97.3%	94.9%
Nodes with path to global optimum	92.5%	99.4%	94.8%	96.4%
ILS success rate	87.1%	31.2%	94.4%	98.4%

The variant that considers only comparison operators is denoted by *c*, while the variant that considers both comparison and Boolean operators is denoted by *c* $+$ *b*

In all benchmarks, LONs are rather sparse but present patterns of local connectivity. In fact, the *clustering coefficient*, that is, the average proportion of transitive closures among the neighbours of a vertex, is always around four orders of magnitude higher than the overall *network density*. That is, connections between nodes that already share a neighbour, are orders of magnitude more frequent than connections in general, which could be explained by the fact that the LON graph displays the traces of iterated local search trajectories.

However, the great majority of these local connections happen on the plateaus that are clearly visible in Figs. 4, 5, 6, 7. Indeed, considering the sampled non-deteriorating moves, more than $99\%$ of the times a transition out of a local minimum leads to another local minimum with the same fitness value. That applies to both problems and both mutation operators subsets.

These plateaus at the LON level are also known as meta-plateaus. Let us note that, in general, meta-plateaus do not necessarily indicate plateaus at the solution level. This is because the standard fitness landscape model considers a single neighbourhood relation *N* while the LON model considers at least two neighbourhoods: one for the definition of local optima (*N*) and another for the edge transitions. It follows that two connected solutions of same fitness in a LON may not be part of the same plateau in the underlying landscape defined by *N*. However, in the present study, the same neighbourhood is used for both the hillclimber and mutation, therefore blurring the difference between a plateau at the landscape level and a meta-plateau at the LON level.

The triangle program LONs are made up of 6 (fitness 0–5) large plateaus relatively well-connected between each other—and there is a tiny fitness 6, easily escapable, plateau when both comparison and Boolean operators are considered (Fig. 5). When mutations on Boolean operators are introduced, more ILS runs get stuck at fitness 2 and are not able to progress to the global optima level. The ILS success rate, i.e., the proportion of runs that reach a global optimum, is measurably lower (87 vs. 31%).

For the tcas program, the maximum observed fitness in the networks is 264. For both programs, we thus have plateaus that are well below the maximum fitness of 14 and 1578. Since random, not locally-optimal solutions, are often close to those maximum values, this indicates that it is fairly easy to improve the solution fitness with a simple hill-climber, at least initially.

The tcas program LONs display more difference between them. Perhaps surprisingly, the variant that only considers comparison operators shows fairly well-defined plateaus—the three main ones have fitness 0, 144 and 264. This may be an artefact of some interaction between mutations. The study of these interactions is beyond the scope of this paper but seems to be an interesting area for future research. The variant with both comparison and Boolean operators shows more “steps” along the different runs and, therefore, less well-defined plateau structures. This potentially means that finding improving solutions, and ultimately a global optimum, is easier. Whilst the ILS success rate for both variants is quite high, there is a marked improvement for the second variant (from 94 to 98%).

Let us observe that there is a high number of global optima, i.e., solutions that are test-equivalent to the original programs. This may mean that the programs are quite robust—we have not tested this hypothesis—or that the test suite does not provide enough coverage.

In terms of global connectivity, we can observe that a path between any pair of nodes is not always present, even if we disregard the direction of the edges. That is, the networks break down into a number of weakly-disconnected components, especially when the larger search space of comparison and Boolean operators is considered. Nonetheless, more than $92\%$ of the local minima we observed belong to a single, largest connected component (Table 2). Moreover, a similar high fraction of all local minima lie on paths that could eventually descend to a global optimum.

By following the steepest descent directions on the LON, we can also detect the presence of multiple attractors with no non-deteriorating transitions around them, i.e., dead-ends for the search. Their number is indicative of the *multi-funnel* global structure of the landscapes [26, 28], and may directly relate to the empirical problem hardness from the point of view of an Iterated Local Search [27]. Among the four benchmark instances, the one with the lowest success rate also has multiple sub-optimal attractors. Indeed, almost all its local minima have access or belong to the funnel containing the global optima, but we hypothesise that, given the ILS stopping criterion, the actual success rate might depend on how easy it is for the search to find exits across plateaus and gain access to better (lower) fitness levels within the budget of function evaluations. As it can be visually appreciated on Fig. 5, the “hardest” instance is also, notably, the one with fewer such connections across the lowest fitness levels.

More generally, the notion of difficulty we consider is based on how easy it is for the algorithm to reach a global optimum. This is influenced—for both the mathematical object that is the LON and its visualisation—by the number of paths that lead to global optimum. Another factor associated to difficulty is whether an algorithm will remain stuck in a local optimum and whether there are regions of the landscape that tend to concentrate those deceptive solutions. A quick visual assessment for difficulty can therefore be to observe the connectivity patterns, or absence thereof, leading to the global optima or to local optima that cannot be escaped. The link between different network metrics and search difficulty has been quantified in other contexts, for instance in [27, 33]. The visualisations presented here provide an immediate translation of the network objects, revealing different connectivity patterns. In the future, it would be interesting to assess how researchers and practitioners in the field of local search search and metaheuristics interpret these visualisations and whether that relates to their intuitions on search difficulty and the objective metrics that can be computed on a landscape.

### Influence of subsamples on visualisations

In general, search landscapes, or even local optima networks, for non-trivial problem instances cannot be enumerated. Sampling is therefore required. Furthermore, the amount of information that can be visually represented in a coherent manner is also limited. In addition, the larger the number of points, the more it becomes computationally expensive to generate a layout. In our experience, we can usually sample networks at a much finer granularity than what can be represented visually on print medium or via non-interactive representations on screen that are discussed here. This means that one or more subsamples from the initial sample are examined. We look into some of the issues of subsampling here.

The networks that were considered in the previous subsection are based on specific subsamples. In the current subsection, we assess how subsampling at the same level and at a higher level influences the visualisations.

**Fig. 8:**
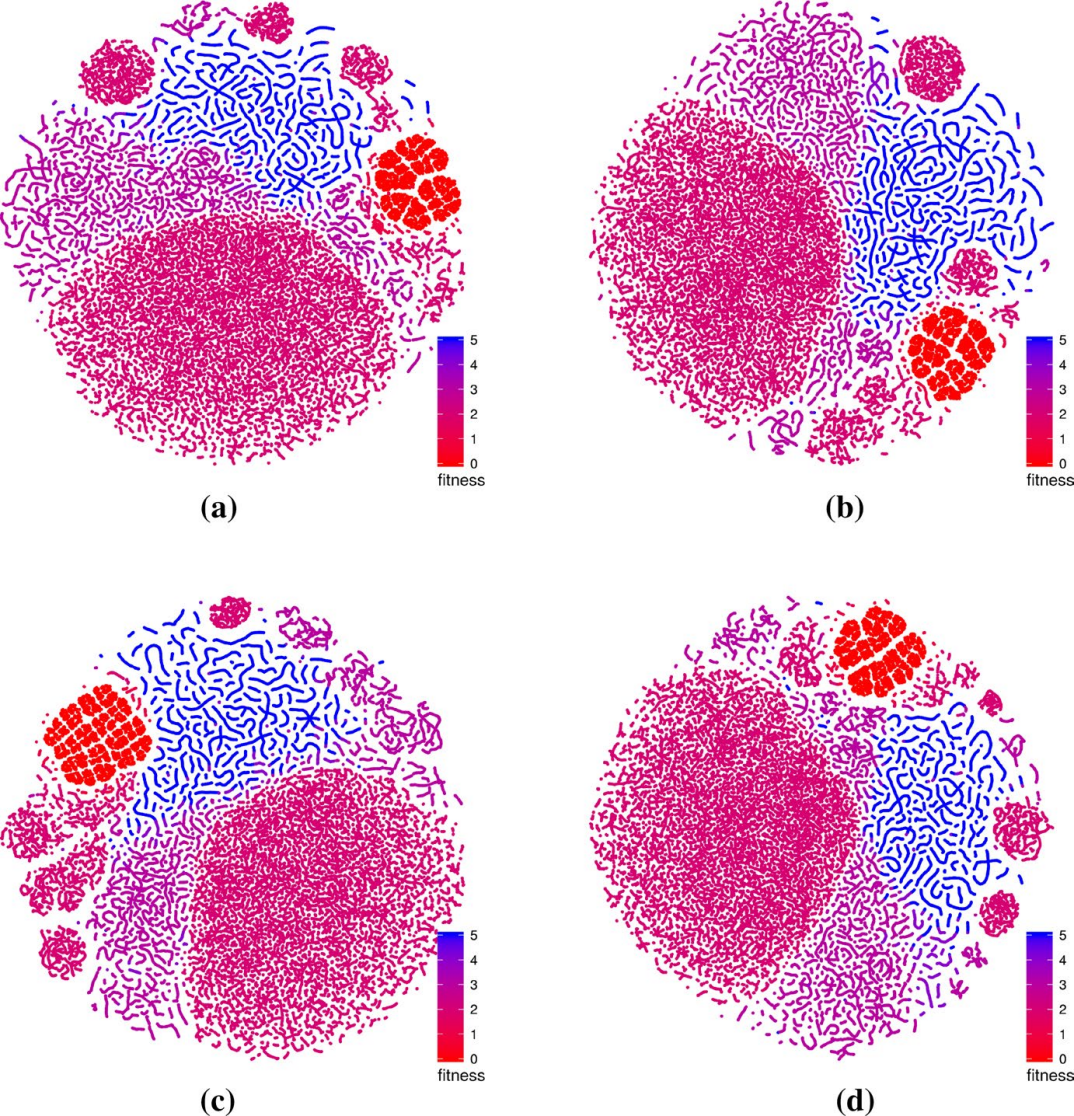
t-SNE layouts for four subsamples of the large sampled LON for the triangle program with comparison operators only. The four sets of points exhibit similar structural patterns and differ from each other mostly in terms of mirroring or rotation. **a** Triangle—subsample 1—runs 1–100, **b** triangle—subsample 2—runs 101–200, **c** triangle—subsample 3—runs 201–300, **d** triangle—subsample 4—runs 301–400 (Color figure online)

**Fig. 9:**
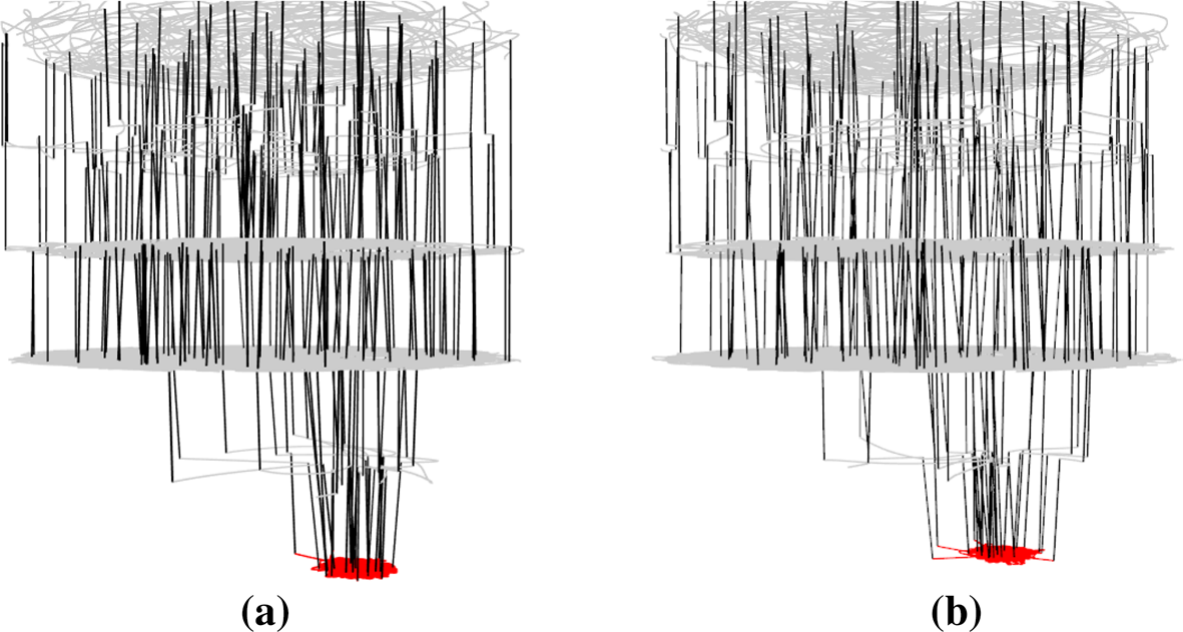
3D view of the force-directed layouts for two subsamples of the large sampled LON for the triangle program with comparison operators only. The two networks are very similar. **a** Triangle—subsample 1—runs 1–100, **b** triangle—subsample 2—runs 101–200 (Color figure online)

**Fig. 10:**
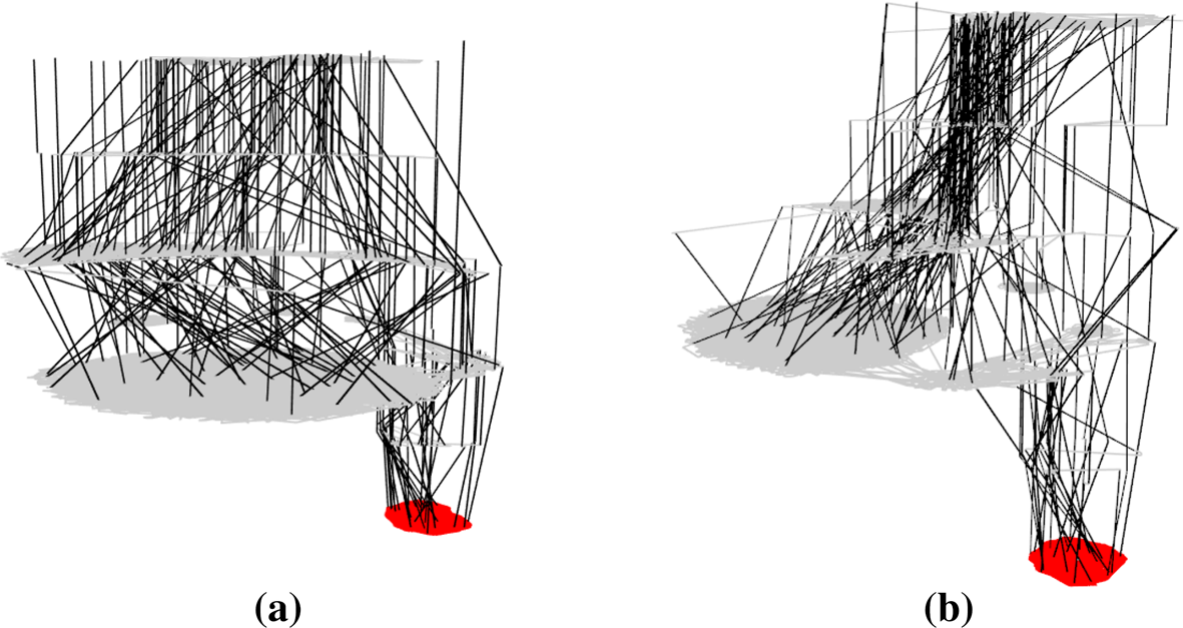
3D view of t-SNE layouts for two subsamples of the large sampled LON for the triangle program with comparison operators only. The two networks appear to be quite different despite using very similar layouts (Fig. 8a, b). This is mainly because of different viewing angles. **a** Triangle—subsample 1—runs 1–100, **b** triangle—subsample 2—runs 101–200 (Color figure online)

Figures 8, 9 and 10 present subsamples of the triangle program where only mutations of comparison operators are considered. Each subsample consists of a different set of 100 runs of 1000 iterations each. The figures show, in order, the t-SNE 2D layouts, force-directed 3D layouts and t-SNE 3D layouts of the subsamples. These are visually very similar. The t-SNE 2D layouts (four of them are shown in Fig. 8) exhibit similar local and global structures across the subsamples (modulo rotation and reflection symmetry). The force-directed 3D layouts (two of them are shown in Fig. 9) are almost indistinguishable at first glance, however the t-SNE 3D layouts (two of them are shown in Fig. 10) appear more different. This is due to different viewing directions—they are based on the t-SNE 2D layouts which are similar. These observations point to the relative robustness of the visualisations for this subsampling level. Similar observations can be made for the other networks but are not visualised here due to space constraints.

**Fig. 11:**
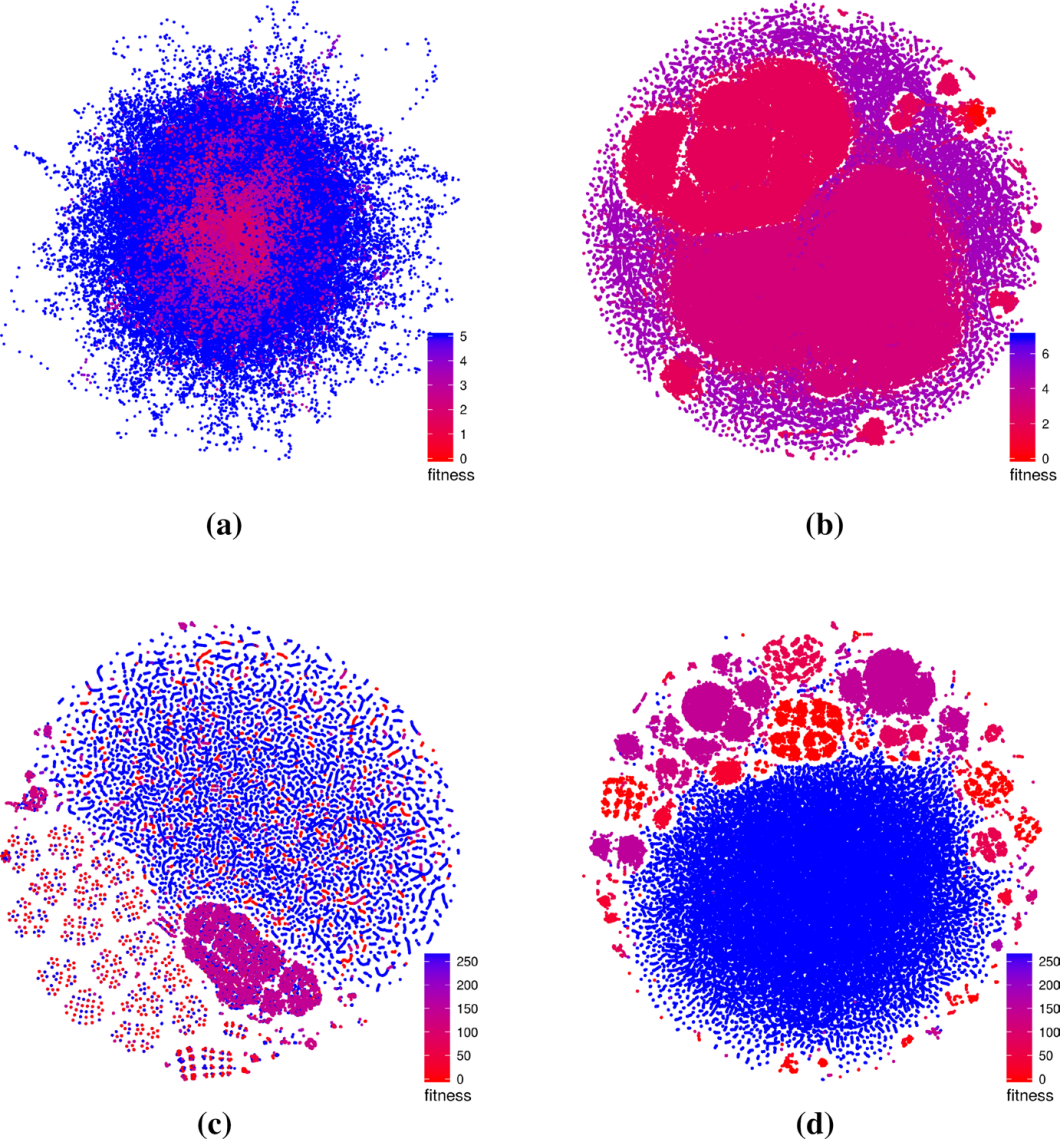
t-SNE layouts for larger subsamples (1000 runs of 1000 iterations) for the four program-operator combinations. The plots exhibit a number of differences with the ones generated using a smaller subset in Figs. 4, 5, 6, 7, in part because of the higher point density, but also because the ratio of solutions across fitness levels is different. **a** Triangle—comparison operators—599,340 solutions, **b** triangle—comparison and Boolean ops—562,272 solutions, **c** tcas—comparison operators—84,967 solutions, **d** tcas—comparison and Boolean ops—322,638 solutions (Color figure online)

Figure 11 considers larger subsamples of 1000 runs of 1000 iterations for each of the four benchmarks and presents them as 2D t-SNE scatterplots. The number of solutions is indicated in the figure’s captions. Our current R implementation is unable to scale to generate 3D plots of such large networks. The scatterplot visualisations for these larger subsamples are naturally denser than for smaller subsamples because of the larger number of points displayed. There are some notable differences between the larger subsamples and their smaller counterparts. This is especially true for the triangle program where only comparison operators are mutated (Fig. 11a). In this case, the two visualisations are completely different and this highlights that any interpretation of the landscape structure visualisations needs to be carefully considered. One of the reasons for the marked contrast is the difference in the distribution of fitness across both subsamples: notably, the proportion of globally optimal solutions (red) in the smaller subsample is much higher than in the larger subsample (4275/64876 vs. 9211/599340 or 6.6 vs. 1.5 %). This difference stems from the fact that the search algorithm is meant to find good solutions, and global optima in particular, and that there is a fixed number of relatively easily discoverable global optima. It is therefore likely for multiple runs to end up discovering the same very good solutions. However, the solutions discovered during the initial part of the search process will almost surely be different for each run as each starts from a totally random solution. When the subsampling size increases, it is much more likely that more poorer quality solutions will be discovered than new global optima, thus explaining the different ratios.

The difference is less notable for the other benchmarks. For the triangle program with mutations of both comparison and Boolean operators, the more local structures are lost because of the higher point density but there is still a similar segregation of points based on fitness. It is interesting to observe that for the two tcas variants, the structures are visually largely similar, especially for lower fitness levels. This is partly due to the lower number of points generated in the tcas samples which reduces the blurring effect that large numbers of points produce when plotted in a restricted area.

### Comparing search techniques

The sampling algorithm naturally biases the networks that are generated. It is therefore interesting and useful to compare and contrast samples generated by different algorithms, and their visualisations. We consider here the networks generated by the Hybrid GA detailed in Sect. 4.2 and Table 3 presents some of their key metrics.

The samples for the Hybrid GA aggregate 1000 runs of 50 generations with a fixed population size of 400, a crossover probability of 0.5 and no mutation. This crossover probability means that roughly 200 new solutions may be created per generation, and thus 10000 per run. The samples for the ILS considered previously aggregated 1000 runs of 10000 iterations each. Naturally, these two sampling techniques produce samples with different characteristics. Notably, the number of nodes and edges generated is higher for the Hybrid GA, especially for tcas with mutations across both comparison and Boolean operators. This difference also translates in terms of number of global optima found—or programs that pass all test cases—except for the triangle program with mutations only on comparison operators where the numbers are the same. In fact, in this scenario both algorithms generated the same set of global optima. Because of the computational complexity we are not able to assess if this is the complete set of globally optimal solutions but it is a possibility. Network density and clustering coefficients are mostly similar across the two sampling techniques. The Hybrid GA samples exhibit lower neutrality, as measured by the neutral degree, which is expected because the ILS has the tendency to explore several solutions in a plateau before being able to escape, which is generally not the case for crossover. A marked difference is in the number of connected components which is drastically higher for the Hybrid GA because it is not a trajectory based method and it employs selection in the parent population. Nevertheless, the relative size of the largest connected component is always well above $90\%$ of all the local optima encountered. Another major difference with the ILS samples is the percentage of nodes with a path to the global optimum which is much lower for the Hybrid GA. The Hybrid GA samples also exhibit a significant difference in terms of success rate, although both variants of the triangle program remain harder to solve across both algorithms. This suggests that the hybrid approach may be better than ILS for genetic improvement.

**Table 3:** Network characteristics and Hybrid GA performance

Program	triangle.c		tcas.c	
Variant	c	c $+$ b	c	c $+$ b
No. of nodes	4,926,235	6,403,728	351,506	6,247,216
No. of edges	14,880,835	19,702,854	7,461,101	12,286,374
No. of global optima	9216	1,142,194	22,824	5,826,627
Network density	${{6.1}}\times 10^{-{7}}$	${{4.8}}\times 10^{-{7}}$	${{6.0}} \times 10^{-{5}}$	${{3.1}} \times 10 ^ {-{7}}$
Clustering coefficient	${{1.7}} \times 10 ^ {-{1}}$	${{2.9}} \times 10^{-{3}}$	${{9.1}}\times 10^{-{2}}$	${{1.4}} \times 10^{-{3}}$
Neutral degree	84.9%	88.6%	95.0%	97.1%
No. of connected components	6527	6532	4179	5387
Relative size of largest conn. comp.	99.4%	99.6%	94.7%	99.6%
Nodes with path to global optimum	36.6%	26.1%	37.2%	35.2%
Hybrid GA success rate	74.2%	76.5%	100%	100%

The variant that considers only comparison operators is denoted by *c*, while the variant that considers both comparison and Boolean operators is denoted by *c* $+$ *b*

**Fig. 12:**
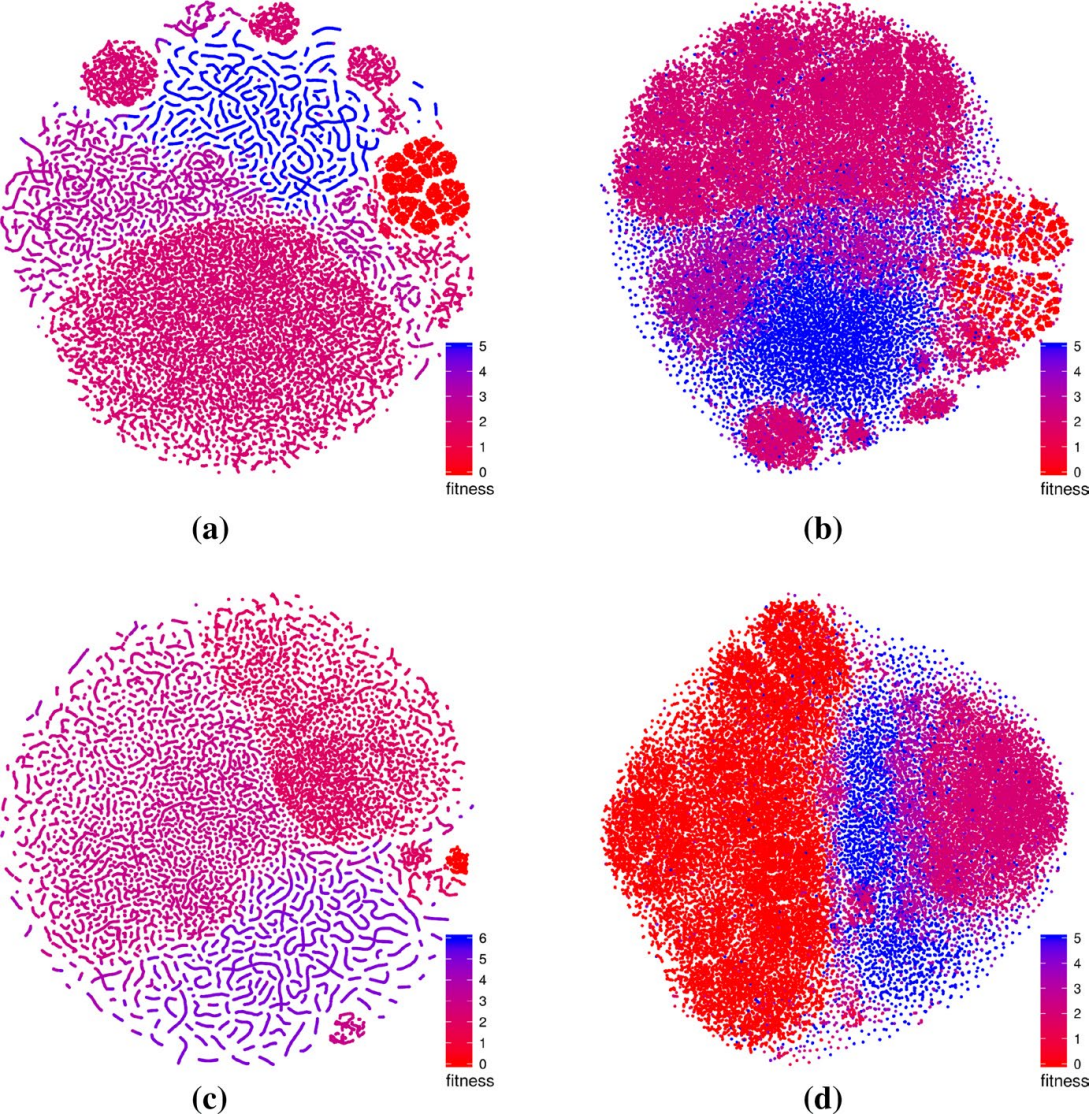
Comparison of subsampled solutions for the hybrid algorithm and the ILS triangle landscapes. Clustering of solutions with similar fitness is observed in both ILS and hybrid GA samples. However, the worm-like structures only appear for the ILS because they are artefacts of the search procedure. **a** ILS—comparison operators—64,876 solutions (100 runs), **b** Hybrid GA—comparison operators—70,406 solutions (12 runs), **c** ILS—comparison and Boolean ops—56,837 solutions (100 runs), **d** Hybrid GA—comparison and Boolean—58,198 solutions (6 runs) (Color figure online)

**Fig. 13:**
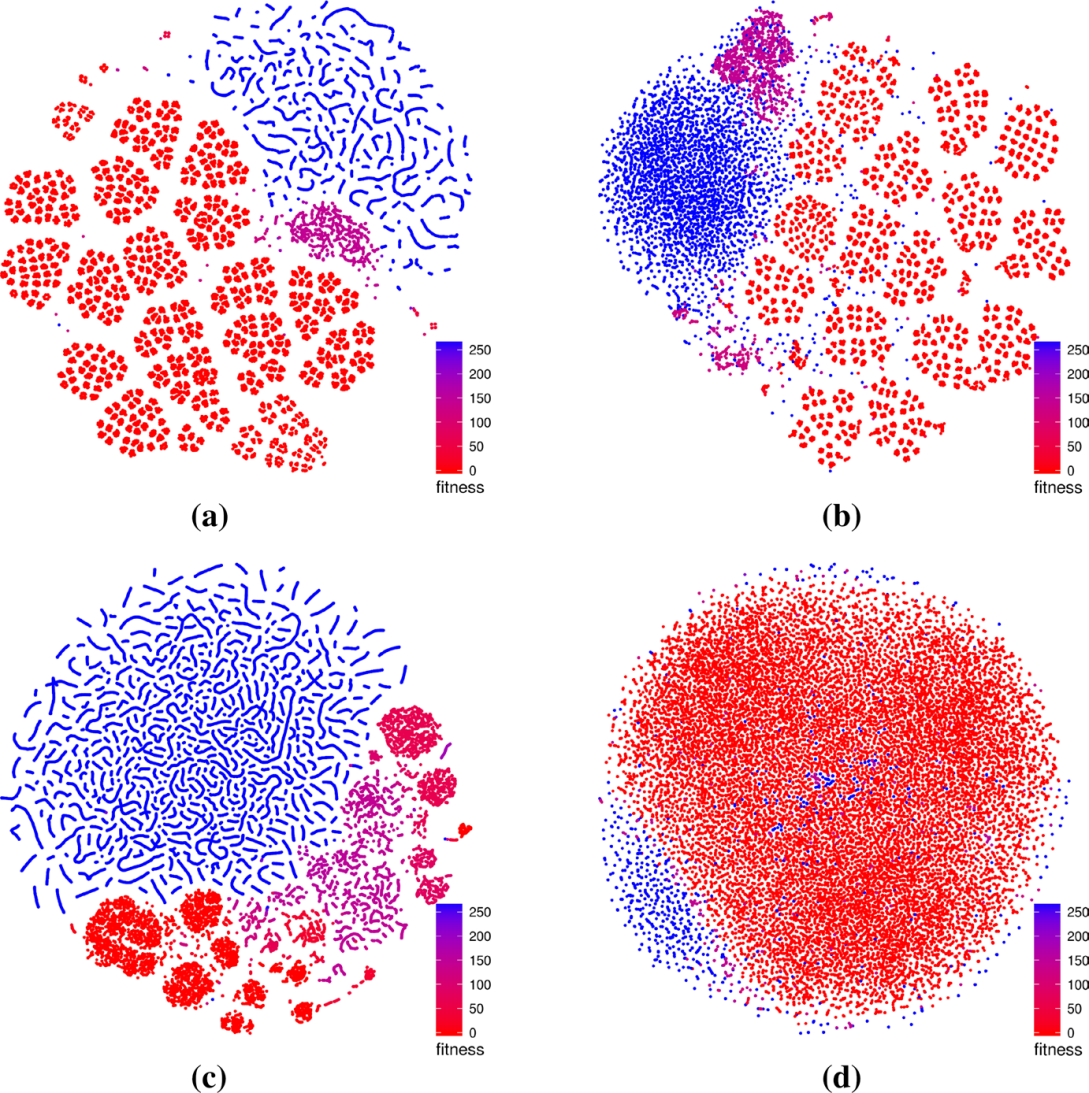
Comparison of subsampled solutions for the hybrid algorithm and the ILS tcas landscapes. Solutions with similar fitness are mostly clustered together. When both comparison and Boolean operators are considered, the hybrid GA is able to traverse the search space more efficiently and finds a proportionally large number of global optima. **a** ILS—comparison operators— 25,533 solutions (100 runs), **b** Hybrid GA—comparison operators—27,537 solutions (100 runs), **c** ILS—comparison and Boolean ops—34,201 solutions (100 runs), **d** Hybrid GA—comparison and Boolean—30,400 solutions (12 runs) (Color figure online)

Figures 12 and 13 highlight the differences in terms of t-SNE scatterplot visualisations for the triangle and tcas programs respectively. The ILS visualisations reuse the same 100 run subsample as seen previously while the number of runs per subsample of the Hybrid GA, indicated in the caption of each figure, is chosen to roughly match the number of solutions of the ILS subsample. This is in order to stay within a reasonable and comparable number of points.

Since t-SNE takes the dissimilarity between points into account when assigning their coordinates, it is reasonable to compare each sample type side-by-side. If both sampling methods returned roughly the same set of solutions or sets of solutions with very similar characteristics, one would expect the dissimilarities to be roughly equivalent and thus translate to similar visualisations. Here we can see that the two methods yield relatively similar visualisations for the benchmarks that only consider comparison operators. Globally optimal solutions, in particular, exhibit the same clustering patterns and solutions of equivalent fitness are grouped together indicating that they occupy specific areas of the search space. One major difference are the worm-like structures in the ILS sample—artefacts of this single point trajectory sampling methodology—which do not appear in the Hybrid GA samples.

In comparison, the variants that explore the mutations of both comparison and Boolean operators are markedly different, although there is still a differentiation in the areas occupied by various fitness levels. One key difference is the number of globally optimal solutions which is much greater for the Hybrid GA and which therefore influences the layouts produced. One possible explanation for this difference is that there may be disjoint plateaus of globally optimal solutions—or plateaus that are extremely large—of which the ILS is able to explore a smaller subset that the Hybrid GA. In addition, we observed that the Hybrid GA converged very quickly to the globally optima solutions, usually within 10 generations or fewer, strongly suggesting that the landscapes should be quite different.

We do not show any 3D visualisations for the Hybrid GA because the number of crossing edges tends to create massive hairballs that do not provide significant useful information. One potential way to deal with this issue in the future would be to use *edge bundling* [11] which routes related edges along similar paths.

**Fig. 14:**
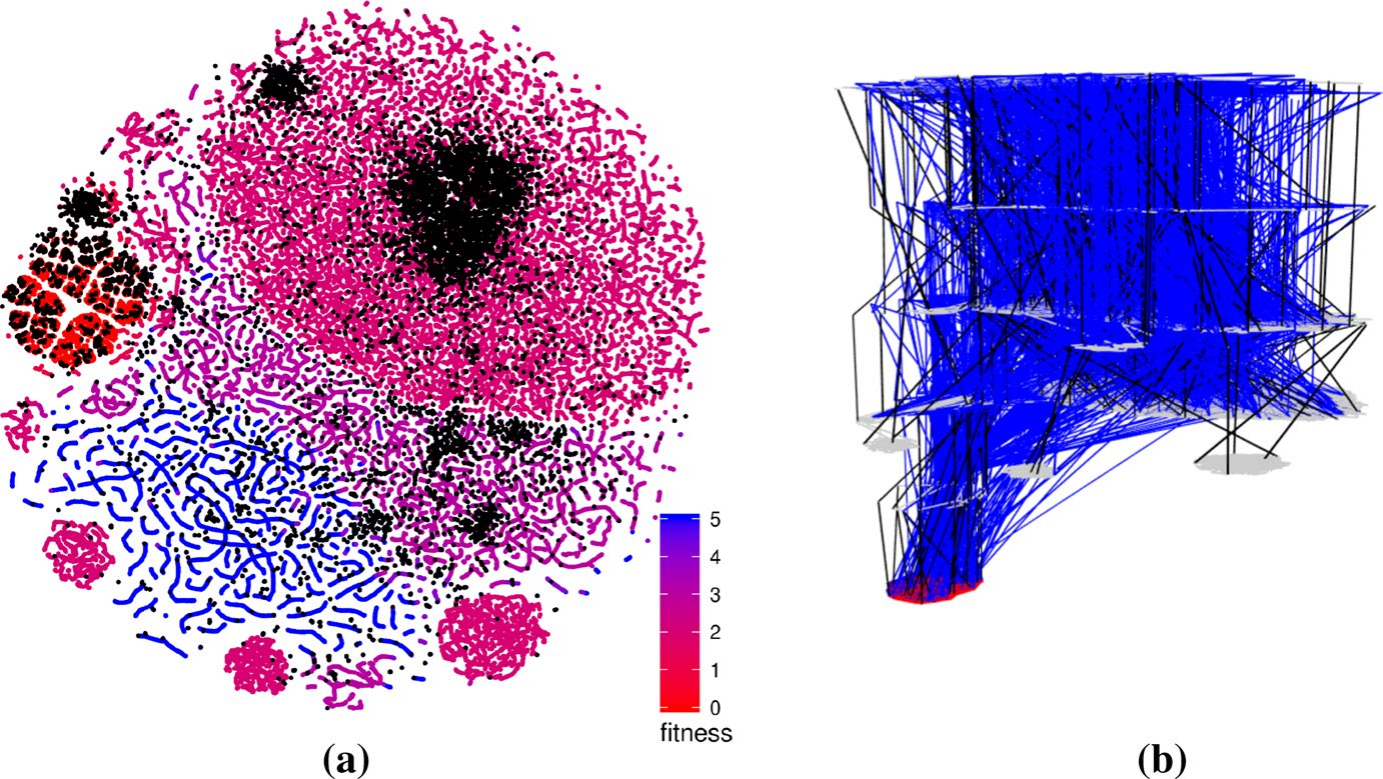
Combination of subsampled LONs for the hybrid algorithm and the ILS for the triangle program landscape considering comparison operators only. The points in black in the scatter plot and the edges in blue in 3D view belong to the sample of the hybrid algorithm. **a** t-SNE layout, **b** 3D view of t-SNE layout (Color figure online)

In addition to side-by-side comparisons, the t-SNE layouts can be used to overlay two sets of points to highlight similarities and differences. In this scenario, both sets of points are merged and a single layout is computed. Figure 14 shows such an overlay, with 100 runs of the ILS in the background and 2 runs of the Hybrid GA on top. We only show a smaller subset of the Hybrid GA points because otherwise they would cover the ILS points. This serves to highlight the fact that similar areas of the search space are explored by both algorithm. In an interactive context, the overlay could be manipulated dynamically to reveal different levels of information. The 3D view is less informative because of the large number of crossing edges.

## Conclusion

Understanding the structure of search landscapes is essential in order to develop efficient algorithms to solve hard combinatorial optimisation problems, and genetic improvement problems in particular. While different approaches of visualising search trajectories and landscapes have been proposed, there have been few attempts to consider the global structure of the landscapes. Local Optima Networks allow us to fill this gap by coarsening the representation of the landscape to local optima. In this paper we have proposed using the t-SNE algorithm to generate layouts for those networks, thereby teasing out different structures at different levels. These plots bring the landscape metaphor to life in, what we believe is, an intuitive and almost tangible way.

We found that the visualisations, together with network metrics, could describe different features and properties of the landscape, for instance, neutrality and the existence of multiple pathways throughout the networks to reach globally optimal solutions. One striking observation was the large number of solutions that pass all the test cases of the benchmark problems. This raises many additional questions in terms of the suitability and coverage of the test suite and on the semantics of the test-equivalent mutants. This will need to be addressed in future work.

In this paper, we have also attempted to assess the quality and robustness of the visualisations when considering various subsamples. Our results show that care needs to be taken when interpreting the visualisations. Subsamples of the same landscape, generated with the same parameters, and representing sets of points of roughly the same size were very similar but exhibited some mirroring or rotation of the patterns appearing within the visualisations. When subsamples of very different sizes were considered, quite similar visualisations were produced in some cases and quite different ones in other cases. This highlights the importance of considering samples or subsamples with different characteristics in order to obtain a broader picture of the search landscape. The visualisations also allow for the comparison of the different search methods, as we have shown.

Genetic improvement approaches rarely make use of Iterated Local Search or of hybrid GAs, yet, we have shown that these techniques are able to find programs that pass all test cases. This observation should be an encouragement to further explore these algorithms in the context of GI.

Our tools rely on metaheuristics which can be instrumented in order to sample a fitness landscape. They can be used in multiple contexts and across a diverse set of problems. The visualisations are created with off-the-shelf software in the form of R and Python scripts and additional free libraries. However, these currently have rendering limits which will need to be overcome in future work. As we mentioned earlier, edge bundling could be a valuable addition to the visualisations in order to reduce edge clutter. From a technical standpoint, the rendering engine that drives the rgl package in R is fairly limited in terms of features and performance. One alternative, while still keeping the possibility of programmatically manipulating the network objects, could be to use a dedicated 3D rendering package such as Blender which provides a Python interface.

In addition, this paper has not considered interactive visualisations which could prove very useful to end users. One instance of this could be allowing certain paths within the network to be isolated and observed as standalone objects. Another aspect could be allowing for the comparison of test-equivalent mutants: contextual information about the differences in the source code of the mutants could be displayed, as well as highlighting which solutions are within some predetermined edit distance from the solution currently being observed.

## Data Availability

Data and figures from this paper are available from the Stirling Online Repository for Research Data (http://hdl.handle.net/11667/120).

## Footnotes

1 http://sir.unl.edu/content/sir.php.
